# Dr. John D. Zwetsch

**Published:** 1916-06

**Authors:** 


**Dr. John D. Zwetsch,** Cleveland Homoeopathic 1882, was killed in an automobile accident near Gowanda, where he had practiced for many years, May 6.

